# Psychometric Properties of the Chinese Version of the Psy-Flex Among Parents of Children with Autism Spectrum Disorder

**DOI:** 10.1007/s10803-024-06610-0

**Published:** 2024-10-27

**Authors:** Si Ni Li, Wai Tong Chien, Stanley Kam Ki Lam, Yuen Yu Chong, Andrew T. Gloster

**Affiliations:** 1 The Nethersole School of Nursing, Faculty of Medicine, The Chinese University of Hong Kong, Hong Kong, China; 2 Division of Clinical Psychology & Intervention Science, Department of Psychology, University of Basel, Missionsstrasse 62-64, 4055 Basel, Switzerland

**Keywords:** Autism spectrum disorders, Parents, Psychological flexibility, Psychometric evaluation

## Abstract

This research aimed to translate the original English version of the Psy-Flex, a scale of psychological flexibility, into Chinese and to test its psychometric properties among parents of children with autism spectrum disorder (ASD). Two phases were conducted: (1) translation from English to Chinese (Psy-Flex-C), followed by a semantic equivalence evaluation between two versions, a pre-test, and an evaluation of the Psy-Flex-C in terms of face validity with 20 parents of autistic children, and content validity of the Psy-Flex-C with eight experts. (2) A cross-sectional study with 248 parents of autistic children was conducted for validation, and a subgroup of 50 participants was randomly selected to assess the test–retest reliability at a 2-week interval. The Psy-Flex-C showed satisfactory semantic equivalence with the original version and demonstrated adequate internal consistency (Cronbach’s α = 0.84) and test–retest stability (weighted kappa statistic = 0.88). Concurrent validity was supported by a moderate correlation between the Psy-Flex-C and the Comprehensive Assessment of Acceptance and Commitment Therapy Processes (Pearson’s r = 0.54, *p* < 0.01). The Psy-Flex-C showed a significant mean score difference between parents with high and low parenting stress (t = 5.43, *p* < 0.001). Similar to the original scale, confirmatory factor analysis showed the best fitting one-factor structure of the Psy-Flex-C (X^2^/df = 1.62, *p* = 0.13, RMSEA = 0.05, GFI = 0.99, CFI = 0.99, TLI = 0.98, SRMR = 0.023). The Psy-Flex-C can be a reliable and valid instrument to self-report psychological flexibility in parents of children with ASD. Future research is recommended to test the Psy-Flex-C using diverse samples from different cultures and contexts to enhance its generalizability.

## Introduction

Autism spectrum disorder (ASD) is a heterogeneous neurodevelopmental condition with a specific combination of deficits in social communication and interaction, as well as repetitive, restrictive, and inflexible patterns of interests, behaviours, or activities beginning early in life (World Health Organization, 2018). According to the Global Burden of Diseases, in 2019, ASD impacted approximately 28 million individuals (Solmi et al., 2022) and nearly three million children under five years old (Li et al., 2022) worldwide. Approximately 35% of children with ASD exhibit intelligence disability (defined as an intelligence quotient score ≤ 70 and experienced difficulties in adaptive functioning) (Maenner et al., 2021), and 92% suffer psychiatric comorbidities such as attention-deficit/hyperactivity disorder as well as other emotional and behavioural problems (Brookman-Frazee et al., 2018), leading to the propensity for long-term dependence on their parents and other family caregivers.

Parenting children with ASD is a challenging experience, particularly in mainland China, where the healthcare, educational, and social services for ASD families are in their infancy (Liu & To, 2022). Due to constraints in public services in mainland China, most ASD-related interventions have been provided by grassroots organizations funded by parents of children with ASD or by individuals with inadequate professional knowledge about ASD and insufficient training to implement ASD interventions (Liu & To, 2021). For the same reason, these parents are obligated to fulfil dual roles as caregivers (e.g., coping with their children’s ASD-related conditions and comorbidities) and as partners in multiple, complex, intervention programs (e.g., parent-mediated interventions) (Kurzrok et al., 2021). These parents need to devote an enormous amount of time, effort, energy, and money to support and nurture their children’s life development and daily living (Marsack-Topolewski & Church, 2019). As a result, parents of children with ASD frequently encounter emotional difficulties (manifesting as distress, grief, denial, and other emotional problems) in accepting and accommodating their children’s ASD-related conditions (Drouillard, 2019). These chronic emotional difficulties not only reduce parents’ ability to sustain daily roles but also increase the potential for developing mental health problems. Previous cross-sectional studies reported that 44% of parents of children with ASD had clinically significant parental stress, 55% had psychological distress, and 34.2% had depressive symptoms (Gatzoyia et al., 2014; van der Lubbe et al., 2022). These emotional difficulties and mental health problems may prevent parents from adapting flexibly to their ASD children’s needs and taking appropriate actions to help their children reach their fullest developmental potential (Moreno Méndez et al., 2020). Furthermore, long-term intensive and rigorous work with their children in everyday caregiving may cause these parents to neglect self-care, which can have negative impacts on their motivation to participate in parental intervention (e.g., parent-focused or parent-mediated interventions) and influence the effectiveness of these interventions (Alvarez et al., 2015). Consequently, this may result in further negative influences on their children’s developmental outcomes (Operto et al., 2021). Given the high prevalence of parental mental health problems and its negative impacts, as well as their emotional difficulties and unmet needs (e.g., neglecting self-care), it is crucial to help these parents care for themselves with openness and acceptance so that, in turn, they can maintain their well-being in caregiving.

Higher psychological flexibility (PF) is associated with lower parenting stress (Fonseca et al., 2020), which enables parents to stay engaged with their ASD children and effectively adapt to the demands of their children and themselves in parenting/caregiving, and it further benefits their children’s health outcomes. PF is defined as an individual’s capacity to accept, cope with, and adjust to difficult situations (e.g., stay open, present, and engaged with their children regardless of unpleasant thoughts, feelings, and physiological sensations) while adapting one’s behaviours based on the situation and personal values (Kashdan et al., 2020). Similar to cognitive flexibility, which was frequently utilized in autism and neuropsychological research and defined as the ability to adapt one’s thinking, cognitive process, and behaviors in response to changing circumstances, PF also involve adaptability and responsiveness to changing circumstances (Aslan & Türk, 2022). However, while cognitive flexibility is more specific to cognitive domains, such as attentional shifting and task switching, PF encompasses a broader range of cognitive, emotional, and behavioral processes (e.g., acceptance, values clarification, and committed action) (Grant & Cassidy, 2022). Previous studies indicated a significantly negative association between psychological inflexibility and mental health, indicating that psychological inflexibility could predict anxiety, depression, and stress symptomatology in mothers of children with ASD (Kulasinghe et al., 2021). Conversely, parents with higher PF show better well-being (Cachia et al., 2016). They are more willing to accept their own experience, redefine the meaning of parenting, and reassess their priorities for their ASD children’s intervention and development, rather than struggling with their negative thoughts and feelings (Byrne et al., 2021). These parents are more likely to adopt adaptive parenting style and behaviors, such as focus on meaningful things (e.g., their children’s motives and intentions as well as self-care) and take value-based actions to better support their children with ASD (Dai & Carter, 2022). Furthermore, parental PF is related to child outcomes (e.g., internalizing and externalizing problems) through adaptive parenting practices (Brassell et al., 2016). Accordingly, it is important to improve parents’ PF using evidence-based interventions, such as acceptance and commitment therapy (ACT).

### Measurements for Psychological Flexibility

However, before designing an effective intervention to enhance the PF of parents of children with ASD, a reliable and valid instrument for evaluating parents’ PF is needed. A few instruments were developed to measure PF or psychological inflexibility, but a few major weaknesses were observed. Firstly, most measures have focused on one to two of the six omains of PF, leaving other domains unassessed. For example, the Acceptance and Action Questionnaire (AAQ; Hayes et al., [2004]) and its second version (Bond et al., 2011) primarily assess acceptance/experiential avoidance and cognitive defusion processes, while neglecting aspects such as self-as-context and committed action (Francis et al., 2016). Similarly, measures like the Multidimensional Experiential Avoidance Questionnaire (MEAQ; Gámez et al., [2011]), its brief version (Gámez et al., 2014), and the Everyday Psychological Inflexibility Checklist (EPIC; Thompson et al. [2019]) mainly targeted experiential avoidance, omitting other five domains of the PF constructs. Secondly, while a few measures such as Comprehensive Assessment of Acceptance and Commitment Therapy Processes (CompACT; Francis et al. [2016]) and the Multidimensional Psychological Flexibility Inventory (MPFI; Rolffs et al. [2016]) were developed to address this limitation and assess all six aspects of PF, they could be a burden to complete due to a relatively large number of items (e.g., the MPFI consisted of 60 items), particularly vulnerable respondents with severe physical or mental health problems. Thirdly, except for the MPFI and Open and Engaged State Questionnaire (OESQ; Benoy et al. [2019a]), no other PF measures specify a time frame for respondents’ reference or consideration in answering the questions. This lack of temporal specificity may introduce recall bias and inaccuracy in the assessment results, as respondents are required to evaluate their entire life events before selecting an appropriate response category (e.g., “often”) to reflects on their experience (Benoy et al., 2019a, 2019b).

In addition, several questionnaires were developed to assess PF in the context of parenting, such as Parental Acceptance and Action Questionnaire [PAAQ; Cheron et al. [2009]), Experiential Avoidance in Caregiving Questionnaire (EACQ; Losada et al. [2014]), Parental Psychological Flexibility Questionnaire (PPFQ; Burke and Moore [2015]), Parental Acceptance Questionnaire (6-PAQ; Greene et al. [2015]), and Parenting-Specific Psychological Flexibility (PSPF; Brassell et al. [2016]). While these tools could be useful in assessing parental PF, several major limitations were noted. First, all the PF questionnaires had only been validated in specific target sample(s); and none of them was validated in parents with ASD. Second, most of these tools (e.g., EACQ [Losada et al., 2014], PPFQ [Burke & Moore, 2015], and PSPF [Brassell et al., 2016]) did not assess all the six processes/domains of PF. Third, some of the measures showed unsatisfactory or uncertain reliability and validity. For example, questionable internal consistency of the PAAQ was reported (Cronbach’s a = 0.65; Cheron et al. [2009]). The stability of 6-PAQ was unexplored (Greene et al., 2015), and the concurrent validity of EACQ was unsatisfactory (Losada et al., 2014). As parents of children with ASD have vulnerable mental conditions and very demanding caregiving, a brief and precise questionnaire (e.g., Psy-Flex) to measure all six domains of PF should be validated among these parents for research and practice use.

### The Psy-Flex

With these limitations in mind, the Psy-Flex was developed to efficiently measure PF (Gloster et al., 2021). The Psy-Flex measures all six PF skills based on the ACT theory through only six items, minimizing the response burden. The strength of the instrument lies in its utilization of a shorter, more specific referent time frame (past one week) to reduce recall biases and inaccuracy. By doing so, it reduces boosting the context sensitivity and serves as an effective outcome measure that accurately reflects changes in response to an intervention. Moreover, the Psy-Flex has been demonstrated to be a reliable and effective tool for assessing PF across different populations and countries (Gloster et al., 2021; Papageorgiou et al., 2021). Specifically, the original English version of the Psy-Flex showed excellent internal reliability for total samples (Cronbach’s α = 0.91) and subsamples of non-clinical individuals (community members: α = 0.90, romantic dyads: α = 0.91) and in- and outpatients diagnosed with depression and anxiety disorders (inpatients: α = 0.78; outpatients: α = 0.97) in Switzerland (Gloster et al., 2021). A one-factor model was confirmed for the Psy-Flex using confirmatory factor analysis (CFA), with an acceptable model fit across all of the subsamples used. Papageorgiou et al. (2021) also validated the Psy-Flex in a Greek-Cypriot sample and reported good psychometric properties. In addition, this measurement could predict a unique variance in well-being and overall symptomatology, thus moderating the relationships between measures of pathology and well-being (Gloster et al., 2021, 2023). Therefore, this brief and clinically useful instrument could help researchers estimate PF in clinical trials to evaluate the effectiveness of ACT-based interventions.

Given that parents of children with ASD have very high psychological distress, a reliable and valid instrument can aid healthcare providers in comprehending these parents’/caregivers’ PF and related distress and finding ways to improve their fewer desirable aspects of self-care and, thus, their parenting. Therefore, this study aimed to translate the English version of the Psy-Flex into the simplified Chinese language and to test the semantic equivalence and other psychometric properties of the translated Chinese Psy-Flex (Psy-Flex-C) among parents of children with ASD in China. The study objectives were to (1) translate the Psy-Flex into simplified Chinese from and back-translate to the original English version and test their semantic equivalence; and (2) evaluate the reliability and validity of the simplified Chinese version in Chinese parents of children with ASD.

## Methods

This study was performed in two phases: (1) translation of the original English version of the Psy-Flex into simplified Chinese and back-translation, followed by an evaluation of the semantic equivalence between the two versions, a pre-test with potential end users (parents), and an evaluation of the face and content validity of the Psy-Flex-C; and (2) an evaluation of the psychometric properties of the Psy-Flex-C in terms of its structure, concurrent, known-group, and convergent validity, as well as internal consistency and test–retest reliability.

### Phase One: Translation, Semantic Equivalence, Pre-test, and Face and Content Validity of the Psy-Flex-C

The revised Brislin translation model (Jones et al., 2001), including back-translation, a bilingual technique, a committee approach, and a pre-test (Brislin, 1970), and Cha’s combined translation technique (Cha et al., 2007) were adopted in the translation process.

The steps of the translation process included the following: (1) the first step was *forward translation*. Two bilingual academic nursing professors independently translated the instrument from the original (English) language into the target (simplified Chinese) language. Through discussion and consensus between the two translators, amendments were made before the Chinese version was ready for the back-translation process. (2) The second step was *back-translation.* Two individuals were invited blindly (without access to the version in the original language) and independently to conduct the back-translation process. One was a bilingual Doctor of Philosophy (Ph.D.) student who was a registered nurse, and the other was a professional Chinese-English translator. Group discussions were performed to compare and discuss any differences between the two back-translations until an agreement was reached. (3) The third step was the *comparison of the original and back-translated English versions.* Our research team invited the author of the original scale to help compare the original English version and its back-translated English version using a four-point Likert scale (1 = ”not appropriate” to 4 = ”most appropriate”). The original author commented on the appropriateness of the translation and confirmed whether the meanings of the statements were consistent with the original scale. Based on the original author’s comments, two translators retranslated items if the original author rated the items ≤ 2. The researcher (first author) then discussed with the two translators to reach consensus on the revised translated items.

The semantic equivalence between the items of the original English Psy-Flex and the Chinese translation was examined to detect any unidentified translation inconsistencies and prevent culture biases towards the original version. The cross-language testing method was used to achieve semantic equivalence (Jones et al., 2001). In this study, 19 Ph.D. students in nursing and two Ph.D. students in psychology were invited to assess the semantic equivalence of the translated version. Each item in the Psy-Flex was rated on a four-point Likert scale (1 = ”not appropriate” to 4 = ”most appropriate”) in terms of equivalence. An item was considered not equivalent if more than 20% of the raters evaluated the item as less than three (Polit & Beck, 2008). The non-equivalent items were revised by re-running the forward and backward translation processes until consensus was reached between the translators and researchers.

The Psy-Flex-C was then pre-tested with 20 parents of children with ASD conveniently recruited from a special education school in Guangdong Province, China, to help the researchers identify potential problems (for example, regarding accessing, understanding, completing, and submitting the online questionnaire) and prepare for testing its psychometric properties (Cha et al., 2007). The response rate and time of the questionnaire completion were recorded and calculated. In addition, face validity was evaluated alongside the process of pre-testing by end users for the same group of participants using face-to-face interviews. Participants were asked to read each item, select a response, and express their thoughts following the think-aloud approach (Willis & Artino, 2013). Probing questions were used to gather their feedback on any difficulties that they had understanding or responding to each item (e.g., “What do you suppose this questionnaire and each item are measuring?”), the items’ relevance to the parenting context (e.g., “Do you think this outcome is crucial for parenting your children with ASD?”), and suggestions for improving the user-friendliness and clarity of the items (e.g., “In your opinion, which items require improvement to increase their clarity and comprehension?”). The clarity of individual items was determined based on the requests by the parents and, thus, the need for explanations. Each interview lasted approximately 15–20 min. The data were coded using a transcription approach (Knafl et al., 2007). Based on the feedback provided by the 20 participants, adjustments were made through discussion among the members of the research team.

The content validity of the Psy-Flex-C was examined by a group of experts (*n* = 8) who were experienced in family care (particularly psychological care) for ASD or who had expertise in ACT knowledge or research. The expert panel consisted of four psychiatrists, two paediatricians, one psychiatric advanced practice nurse, and one psychologist. The panel members were asked to independently rate each item of the Psy-Flex-C using a four-point Likert scale (1 = ”not relevant” to 4 = ”highly relevant”) in terms of its relevance to PF and content clarity. The content validity index (CVI) was examined at the item (I-CVI) and scale (S-CVI) levels. Items rated ≥ 3 were considered acceptable in terms of their relevance to PF measurement. The Psy-Flex-C was regarded as having adequate content validity if the I-CVI was ≥ 0.78 and the S-CVI was ≥ 0.90 (Polit et al., 2007).

### Phase Two: Testing the Psychometric Properties of the Psy-Flex-C

#### Participants and Study Setting

This study was conducted using a convenience sample at four special education schools in Jiangsu, Guangdong, and Guangxi Provinces in mainland China. Participants were recruited if they were (1) over 18 years old; (2) fathers or mothers of school-age (aged between 4 to 12 years; Erikson [1968]) children diagnosed with ASD; (3) living with their children with ASD and were the primary carers responsible for the daily care of their children; and (4) able to complete questionnaires online through a mobile phone or computer. Participants were excluded if they were (1) presenting severe psychiatric symptoms of major or common mental disorders or medical diseases, which could seriously affected their understanding or comprehensibility of the questionnaire and its completion; (2) taking care of one or more other family members with acute/severe/chronic illnesses; or (3) currently caring for their children with ASD who was either currently hospitalized or had a scheduled hospitalization in the coming 1–2 weeks. Based on these study criteria, questions were set before the online questionnaires (the first 1–2 pages) to help the researcher screen eligible subjects.

#### Sample Size Estimation

A priori calculation of sample size was performed. The minimum sample size for CFA was 200 to ensure factor stability (Kyriazos, 2018), which would require the largest sample size in this psychometric testing. In phase one, another 20 parents were recruited for a pre-test by end users and to evaluate face validity. In addition, the evaluation of test–retest reliability required approximately 50 participants randomly selected from the 200 participants in phase two to ensure adequate reliability (Polit, 2014).

#### Instruments

A study specific sociodemographic and clinical data sheet was used to collect information/characteristics of parents (e.g., age, sex, marital status, educational level, employment status, and daily caregiving duration/hours) and children with ASD (e.g., time since the ASD diagnosis, number of comorbid diagnoses, types of training recently attended/attending).

The six-item Psy-Flex is a self-report questionnaire measuring PF by rating on a 5-point Likert scale (5 = ”very often” to 1 = ”very rarely”). A higher score indicates greater PF. The Cronbach’s α was 0.91 for the total sample and ranged from 0.78 to 0.97 for subsamples, demonstrating good internal consistency of the Psy-Flex in both clinical and non-clinical adults (Gloster et al., 2021). Moreover, CFA confirmed a one-factor structure with a good model fit (root mean square error of approximation [RMSEA] = 0.076, comparative fit index [CFI] = 0.98, Tucker–Lewis index [TLI] = 0.96).

The Chinese version of the CompACT was used to test the concurrent validity of the Psy-Flex-C. The Chinese version of the CompACT is an 18-item self-report questionnaire that includes three subscales: openness to experience (CompACT-OE), behavioural awareness (CompACT-BA), and valued action (CompACT-VA) (Chen et al., 2022). The items are answered on a 7-point Likert scale ranging from 0 (“strongly disagree”) to 6 (“strongly agree”). A higher score represents greater levels of PF (openness, awareness, activation). The Cronbach’s α of the Chinese version of the CompACT in Chinese adults was 0.87, indicating good internal consistency (Chen et al., 2022). A three-factor structure, including openness to experience, behavioural awareness, and valued action, was identified through CFA.

The Chinese version of the Parenting Stress Index-Short Form-15 (PSI-SF-15) was used to test the known-group and convergent validity of the Psy-Flex-C. The Chinese PSI-SF-15 (Luo et al., 2021) is a short (15-item) self-report instrument adopted from the original (36-item) PSI-SF and designed to measure three domains in Chinese parents: parental distress (PD), parent‒child dysfunctional interaction (PCDI), and difficult children (DC). The items are rated on a 5-point Likert scale ranging from 1 (“strongly disagree”) to 5 (“strongly agree”). The Cronbach’s α was 0.87/0.86 (mothers/fathers) for the total scale and 0.71/0.72, 0.82/0.78, and 0.79/0.78 for PD, PCDI, and DC, respectively (Luo et al., 2021), confirming its good internal consistency in Chinese parents. The three-factor model with five items per factor was confirmed by CFA with an acceptable model fit and good structural validity (Luo et al., 2021).

#### Ethical Considerations

Approval for this study was obtained from the Secretary of Survey and Behavioural Research Ethics Committee of the Chinese University of Hong Kong (Reference No. SBRE-22-0747) and four special education schools in Jiangsu, Guangdong, and Guangxi Provinces in mainland China. All eligible participants were required to read and understand the information sheet. Research assistants assisted in responding to any questions from the participants. Additionally, the participants were required to sign a consent form before data collection. The research data were accessible only to the researcher, and personal data were encrypted to ensure anonymity. Google Drive was used for secure cloud storage with password protection. After six years from the completion of the study, the personal information will be removed by the researcher.

#### Data Collection

Parents interested in participating in this study were invited to access the online questionnaires by scanning the QR code on the information sheet for recruitment. Before completing the study questionnaires, a series of questions was asked to check the respondents’ eligibility based on the study criteria. Potential subjects were not able to proceed with the study questionnaires if they were identified as ineligible, and a message of appreciation for their interest in this study was provided. The study questionnaires consisted of a sociodemographic and clinical information sheet and the Psy-Flex-C, CompACT, and PSI-SF-15 administered through an online platform (WJX.cn). A group of 50 randomly selected participants from those who provided their contact information completed the study questionnaires again after a two-week interval.

#### Data Analysis

All statistical analyses were conducted using IBM SPSS (version 20) and AMOS (version 28). For reliability, the internal consistency of the Psy-Flex-C was examined using Cronbach’s α and item analysis (corrected item-total correlation [CITC]). Cronbach’s α values of ≥ 0.90, 0.80–0.90, and 0.70–0.80 indicated excellent, good and acceptable internal consistency, respectively (Polit & Beck, 2008). CITC values ranged from 0.40 to 0.70, indicating no redundant items on the scale (Everitt & Skrondal, 2010). The weighted kappa statistic for the Psy-Flex-C’s 2-week retesting interval was calculated to explore its stability (Cohen, 1968), and a result of ≥ 0.70 indicated acceptable stability (Kline, 2000).

To examine the concurrent validity of the Psy-Flex-C, the Pearson product-moment correlation coefficient was used to identify the correlation between the Psy-Flex-C and CompACT scores. Correlation coefficients with values of < 0.25, 0.25–0.50, 0.50–0.75, and > 0.75 were categorized as small, moderate, good, and excellent correlations, respectively (Portney & Watkins, 2009).

By using CFA, the structural validity of the Psy-Flex-C was determined to examine whether its structure was identical to the one-factor structure reported in the original version (Gloster et al., 2021). Maximum likelihood estimation was used to assess the model’s fit. The goodness of fit of the model was tested using the RMSEA, CFI, goodness-of-fit index (GFI), TLI, and standardized root mean square residual (SRMR) (Kline, 2016). RMSEA values ≤ 0.05 indicated an excellent model fit, while values between 0.05 and 0.08 indicated a moderate model fit (Fabrigar et al., 1999). CFI, GFI, and TLI values greater than 0.95 indicated a satisfactory model, while values between 0.90 and 0.95 were acceptable (Bentler, 1990). Moreover, SRMR values ≤ 0.08 indicated an excellent model fit (Hu & Bentler, 1998).

Based on the conceptual proposition of the theoretical model of stress in families of children with developmental disabilities (Perry, 2004) and recent empirical evidence (Fonseca et al., 2020), parents of children with ASD with higher PF exhibited lower levels of parenting stress. To test the known-group validity of the Psy-Flex-C, it was hypothesized that parents with high levels of parenting stress (PSI-SF-15 total score > 45) would have a significantly lower PF score than those without parenting stress or with typical stress levels (PSI-SF-15 total score ≤ 45) by using an independent t test. The convergent validity of the Psy-Flex-C was also determined by calculating the Pearson product-moment correlation coefficient between the Psy-Flex-C and PSI-SF-15 scores.

A 5% level of significance was set for all statistical analyses. To ensure that the assumptions of the parametric tests used were met, the normality, linearity, and homoscedasticity of the Psy-Flex-C, CompACT, and PSI-SF-15 scores were tested using Q‒Q plots, skewness and kurtosis statistics, and a scatterplot (Polit & Beck, 2008). Harman’s single factor test was used to detect common method bias in CFA; that is, no bias can be found if the total variance extracted by one factor is < 50% (Hair, 2010).

## Results

### Phase One: Translation, Semantic Equivalence, Pre-test by End Users, and Face and Content Validity Results

In the Psy-Flex translation process, several inconsistencies in the meanings of a few words or phrases were found. Firstly, two translators noticed a lack of semantic similarity in Item 4, specifically with regards to the translation of “steady core.” This discrepancy led to consultation with a third translator to reach a consensus. Secondly, Item 6 was found to lack consistency and was subsequently revised after discussion between the two back-translators. This was due to the translation of “being engaged” in the original English version being rendered as “engagement”. Thirdly, two items (item 1 and item 3) were identified as inconsistencies by the original author of Psy-Flex during the comparison of the original and back-translated English versions. In Item 1, the word “thoughts” in “Even if I am somewhere else with my thoughts, I can focus on what’s going on in important moments” was turned into “mind”. In Item 3, “hindering thoughts” in “I can look at hindering thoughts from a distance without letting them control me” turned into “inner thoughts.” Based on the original author’s comment regarding the reason for the lack of semantically similar, the third and fourth translators retranslated these items, and the researcher (first author) discussed the revisions with the two translators to reach a consensus on the revised translations. All items of the translated Psy-Flex were rated as ≥ 3 by 95% to 100% of the panel members, and thus, no item needed to be further modified.

Twenty out of 25 parents agreed and completed the pre-test by the end users and the evaluation of face validity (i.e., response rate = 80%; refusals due to lack of interest and time). The average time for these parents to complete the translated Psy-Flex was 4.56 min (SD: 1.03, range: 2.3 to 6.5 min). Ninety-five percent (*n* = 19) of the participants expressed a high level of satisfaction with the overall readability of the Psy-Flex-C. Based on the comments and feedback provided by these 20 end users regarding face validity, a few key words/statements of the translated Psy-Flex were clarified and amended to improve their relevance in terms of translation and culture and to help end users understand these items more easily. For example, item 1, “Even if I am somewhere else with my thoughts”, was amended from its original translated Chinese statement, “即使我和我的思维在其他地方”, to “即使我走神了”. This adjustment enhanced the readability and alignment of the item statements with the Chinese idioms. For item 2, “Being open for experiences”, the original translated statement, “对经验的开放”, was modified to “对经历保持开放的心态” to specify the respondents’ inner perspectives or attitudes.For the content validity of the Psy-Flex-C, all items were rated as ≥ 3 by eight experts; thus, the I-CVI and S-CVI of the Chinese version were both 1. The mean ratings of item relevance of the Psy-Flex-C ranged from 3.50 to 3.88 (out of 4). Therefore, the Psy-Flex-C demonstrated very satisfactory content validity.

The finalized Psy-Flex-C is displayed in Appendix Table 1.

**Table 1:** Socio-demographic characteristics of participants

Characteristics	Mean ± SD or n (%)
*Parents of children with ASD*	
Age (Range: 24 to 55 years)	37.71 ± 5.50
Gender	
Female	201 (81.05)
Male	47 (18.95)
Marital status	
Married	233 (93.95)
Single/separated/divorced/widowed	15 (6.05)
Education level	
≤ Primary	44 (17.74)
Secondary	50 (20.16)
Tertiary	132 (53.23)
≥ Post-graduate	22 (8.87)
Employment status	
Full-time	123 (49.60)
Part-time	25 (10.08)
Unemployment/Retired	100 (40.32)
Spouse’s employment status	
Full-time	181 (72.98)
Part-time	29 (11.69)
Unemployment/Retired	38 (15.32)
Household income (RMB/month)	
≤ 3,000	21 (8.47)
3,001 to 10,000	122 (49.19)
10,001 to 20,000	59 (23.79)
20,001 to 30,000	24 (9.68)
> 30,000	22 (8.87)
Housing types	
Rural self-built house	79 (31.85)
Residential flats	51 (20.56)
Residential apartment	50 (20.16)
Duplex house	15 (6.05)
Villas/bungalow	13 (5.24)
Rent house	40 (16.13)
Household size (number of people)	
2	3 (1.21)
3–4	174 (70.16)
5–6	67 (27.02)
> 6	4 (1.61)
Number of chronic diseases	
0	174 (70.16)
1–2	70 (28.23)
≥ 3	4 (1.61)
Time spent on caring for their autism child (range: 28 to 112 h/ week)	77 ± 26.40
Caregiver support program or intervention attendance	
Yes	117 (47.18)
No	131 (52.82)
*Children with ASD*	
Age (Range: 4 to 12 years)	7.36 ± 2.69
Gender	
Male	199 (80.24)
Female	49 (19.76)
How many years these children have been diagnosed with an ASD (range: 0.1 to 10 years)	4.72 ± 2.57
The level of social communication^a^	
Level 1	73 (29.44)
Level 2	108 (43.54)
Level 3	67 (27.02)
The level of restricted, repetitive behaviors^b^	
Level 1	45 (18.15)
Level 2	189 (76.21)
Level 3	14 (5.65)
Number of comorbidities	
0	82 (33.06)
1–2	146 (58.87)
≥ 3	20 (8.06)
Type of education school currently attending	
Special education	125 (50.40)
Mainstream education	96 (38.71)
Other	27 (10.89)
Time spent Professional behavioral and skills training services (range: 0 to 78 h/ week)	16.46 ± 13.22
Psychotropic drugs intake	
Yes	13 (5.24)
No	235 (94.76)
Number of hospital visits due to ASD in the last year (range: 0 to 8 time[s])	1 ± 1.28
Cost of autism treatment and healthcare services in the last year (range: 0 to 300,000 RMB)	52,101.9 ± 56,899.28

^a^Level 1 = My child can speak in full sentences and engages in communication; Level 2 = My child can use fewer words or noticeably different speech; Level 3 = My child is nonspeaking or has echolalia (repeating words or phrases they hear)

^b^Level 1 = difficulty switching between activities; Level 2 = distress and/or difficulty changing focus or action; Level 3 = great distress and/or difficulty changing focus or action

### Phase Two: Psychometric Properties of Psy-Flex-C

#### Characteristics of the Participants

A total of 397 eligible parents who met the inclusion criteria by completing the online screening questions were invited to participate in the study. Among them, 248 parents (62.47%) of children with ASD completed the study questionnaires. A subgroup of 50 parents was randomly selected from the 248 participants to evaluate test–retest reliability. The baseline sociodemographic characteristics of the participants and their children with ASD are listed in Table 1.

The mean age of the participants was 37.71 years (SD: 5.50, range: 24 to 55 years), and most of them were female (81.00%), were married (94.00%), had a high school education (82.30%), and had a household of ≤ three persons (71.40%). Over half of them were either unemployed (40.30%) or part-time employed (10.10%), having a monthly household income of less than 10,000 RMB (57.70%; approximately 1400 US dollars). The most common housing types were rural self-built houses (31.90%), residential flats (20.60%), and residential apartments (20.20%). More than one-quarter (29.80%) of the participants suffered from chronic diseases, and on average, they spent 77 h (SD: 26.40) per week providing care for their children with ASD.

The mean age of the children was 7.36 years (SD: 2.69, range: 4 to 12 years), and on average, they had been diagnosed with ASD for 4.72 years (SD: 2.57, range: 0.1 to 10.0 years). Most of them were male (80.20%) and had one or more comorbidities (66.93%), limited social interaction/communication (70.5%), and moderate-to-severe restricted and repetitive behaviours (81.80%). Half of them (50.40%) attended special education schools and spent 16.46 h (SD: 13.22) per week receiving behavioural and skills training services from professional medical/rehabilitation centres or schools.

#### Normality, Linearity, and Homogeneity of the Scales

Satisfactory standard skewness and kurtosis scores of the Psy-Flex-C, CompACT, and PSI-SF-15 (range: − 0.25 and 0.13; Table 2 and Appendix Figs. 1, 2, and 3), straight diagonal line Q‒Q plots of these three scales (Appendix Figs. 1, 2, and 3), the cigar-shaped scatterplot between the Psy-Flex-C and PSI-SF-15 (Appendix Fig. 4), and good Harman’s single-factor test results (27.78%; Appendix Table 2) were shown. Therefore, the normality, linearity, and homogeneity of Psy-Flex-C were established.

**Table 2:** Item analysis for the Psy-Flex-C

Item	Corrected item-total correlation	Cronbach’s alpha with item deletion	Distribution of item response n (%)					Stability weighted kappa statistics
			Very often	Often	From time to time	Seldom	Very seldom	
1	0.51	0.83	75 (30.24)	72 (29.03)	75 (30.24)	23 (9.28)	3 (1.21)	0.90
2	0.49	0.83	46 (18.55)	77 (31.05)	85 (34.27)	33 (13.31)	7 (2.82)	0.82
3	0.66	0.80	37 (14.92)	80 (32.26)	92 (37.10)	32 (12.90)	7 (2.82)	0.91
4	0.58	0.82	34 (13.71)	82 (33.07)	90 (36.29)	35 (14.11)	7 (2.82)	0.88
5	0.75	0.79	64 (25.81)	92 (37.10)	68 (27.42)	22 (8.86)	2 (0.81)	0.93
6	0.72	0.79	61 (24.60)	99 (39.92)	64 (25.81)	22 (8.86)	2 (0.81)	0.86
Overall Cronbach’s alpha			0.84					
Overall stability weighted kappa statistics			0.88

**Fig. 1:**
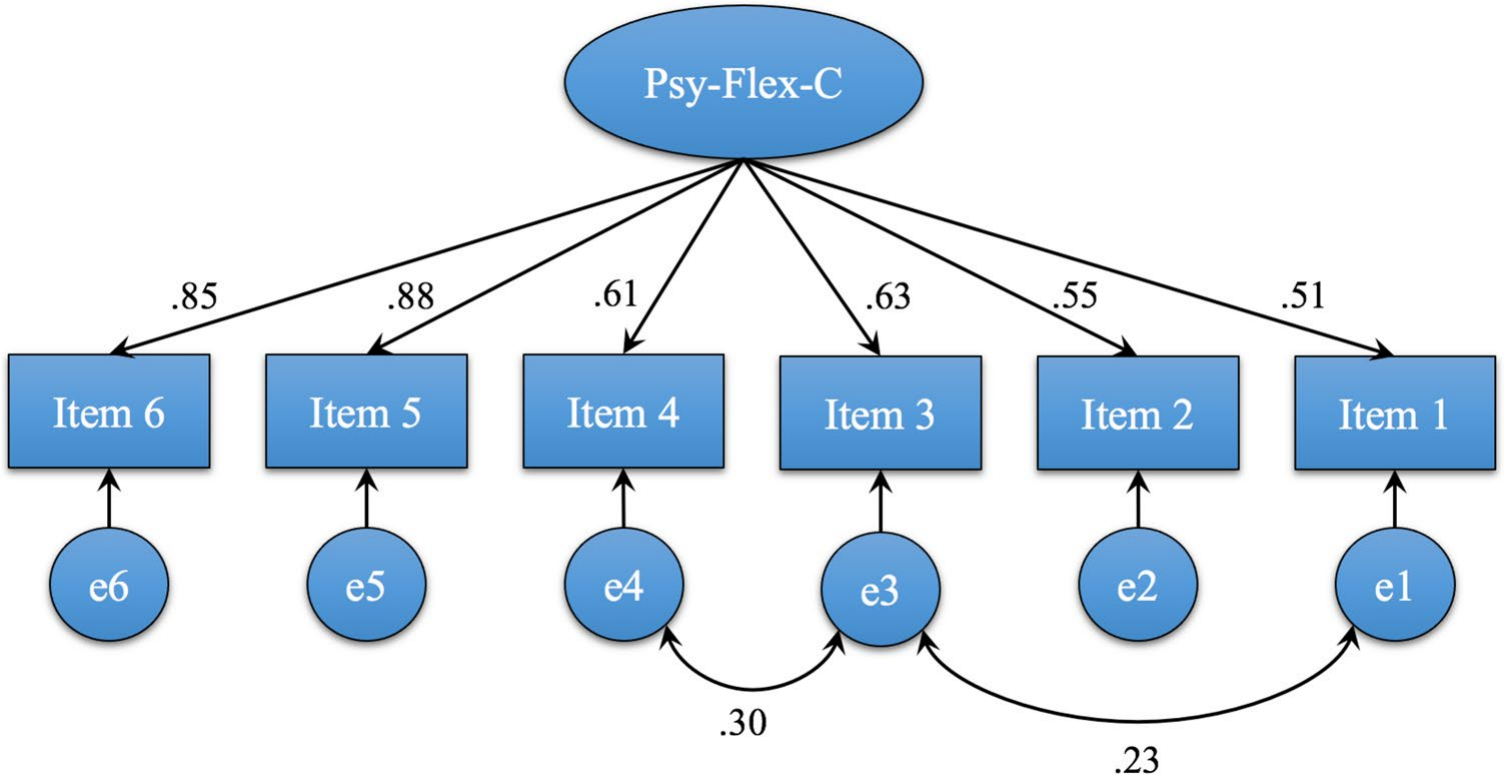
Confirmatory factory analysis of Psy-Flex-C

#### Internal Consistency and Stability of the Psy-Flex-C

The Cronbach’s α of the Psy-Flex-C was 0.84, and the CITC value ranged from 0.49 to 0.75, indicating adequate homogeneity of the construct measured by the scale, with no items requiring deletion (Table 2). For stability, the weighted kappa statistic of the 2-week test–retest reliability (*n* = 50) of the Psy-Flex-C was 0.88 (range: 0.82 [Item 2] to 0.93 [Item 5]), indicating excellent stability (Table 2).

#### Confirmatory Factor Analysis

CFA was conducted for the Psy-Flex-C to validate the rationale of having one factor obtained in the original English version (Gloster et al., 2021). The model was adjusted by constructing paths between the residuals (e.g., e1–e3, and e3–e4 in Fig. 1), and the results indicated that the one-factor model fit was acceptable, with X^2^/df = 1.62, *p* = 0.13, RMSEA = 0.05, GFI = 0.99, CFI = 0.99, TLI = 0.98, and SRMR = 0.023. All items loaded strongly onto the latent factor (0.51 to 0.88; Appendix Table 3), with statistically significant factor loading coefficients (*p* < 0.001).

#### Concurrent Validity

The mean score of the Psy-Flex-C was 21.68 (SD: 4.41, range: 8 to 30), and the mean score of the CompACT was 63.10 (SD: 10.52; range: 37 to 90). There was a statistically significant positive correlation between the two scales (Pearson’s r = 0.54, *p* < 0.01), and significant and moderate correlations were found between the Psy-Flex-C and the three subscales of the CompACT (openness to experience: r = 0.31, *p* < 0.01; behavioural awareness: r = 0.46, *p* < 0.01, and valued action: r = 0.34, *p* < 0.01). These results indicated satisfactory concurrent validity of the Psy-Flex-C with the CompACT.

#### Known‑Group and Convergent Validity

The mean score of the Psy-Flex-C in the parents without parenting stress (PSI-SF-15 ≤ 45) was 22.75 (SD: 4.24; *n* = 160), and that of the parents with parenting stress (PSI-SF-15 > 45) was 19.74 (SD = 3.86; *n* = 88). A significant difference in the Psy-Flex-C score was found between these two groups (t = 5.43, *p* < 0.001), indicating satisfactory known-group validity (i.e., the Psy-Flex-C scores could differentiate between parents with and without high parenting stress).

In addition, the results of convergent validity identified statistically significant and moderate negative correlations between the mean scores of the Psy-Flex and PSI-SF-15 (mean = 41.54, SD = 10.17, r = − 0.42, *p* < 0.01), as well as its subscales (PD: r = − 0.39, *p* < 0.01; PCDI: r = − 0.41, *p* < 0.01; and DC: r = − 0.25, *p* < 0.01).

## Discussion

Our study translated the original English version of the Psy-Flex into simplified Chinese language and examined the reliability and validity of the Psy-Flex-C among Chinese parents of children with ASD. The results suggest that the Psy-Flex-C is an effective and valid measurement tool for PF among these parents. The Psy-Flex-C demonstrates a high level of semantic equivalence with the original scale, satisfactory content and face validity, and consistency in its one-factor structure compared to the original scale (Gloster et al., 2021). It also shows good internal consistency and test–retest reliability, concurrent validity with the CompACT scale, convergent validity with a negative psychological outcome (parenting stress), and known-group validity to distinguish parents with and without high levels of parenting stress.

In terms of semantic equivalence, the Psy-Flex-C is found to be highly equivalent to the concepts and meaning of the items of the original scale. Additionally, the pre-test and evaluation of face validity confirm that the Psy-Flex-C was appropriately translated and understandable by parents with diverse educational levels. Moreover, based on comments provided by eight experts, all Psy-Flex-C items are highly clear and relevant to PF. Therefore, the translated items in Chinese retain the meaning of the original Psy-Flex, and both the English and Chinese versions of the Psy-Flex are content and face valid for assessing PF in parents of children with ASD (Gloster et al., 2021). In addition, similar to the original scale, the satisfactory results of the CITC value and weighted kappa statistic indicate that the Psy-Flex-C has high internal consistency and test–retest reliability to accurately measure PF over time. Therefore, the six-item Psy-Flex-C could potentially be useful for repeatedly detecting PF changes and the development of clients’ ACT skills over time.

Consistent with the original scale (Gloster et al., 2021) and Hebrew version (Gur et al., 2024), the one-factor model is identified through CFA. This finding echoes the ACT model, which indicates that the six interrelated aspects of ACT work together to improve individuals’ PF (Hayes et al., 2006). Moreover, correlated residuals are identified between items 1 and 3 and between items 3 and 4 in the CFA, suggesting a potential correlation between these items. In the ACT model, the six aspects are further categorized into two different change processes: the psychological change process (including contact with the present moment, acceptance, defusion, and self-as-context) and the behaviour change process (including contact with the present moment, self-as-context, values, and committed action) (Hayes et al., 2006). Additionally, based on this model, items 1 (“Being present. Even if I am somewhere else with my thoughts, I can focus on what’s going on in important moments.”), 3 (“Leaving thoughts be. I can look at hindering thoughts from a distance without letting them control me.”), and 4 (“Steady self. Even if thoughts and experiences are confusing me, I can notice something like a steady core inside of me.”) are categorized as the psychological change process (Hayes et al., 2006). These aspects of the psychological change process are all interrelated and interact with each other, hence empowering the behaviour change process. Moreover, all items of the Psy-Flex-C exhibit sufficient factor loadings (ranged from 50.6% to 88.2%). In particular, items 5 (“Awareness of one’s own values”) and 6 (“Being engaged”) demonstrate higher factor loadings (> 80%), indicating a strong association between these behavioral change process items and the latent factor being measured (parents’ PF). This finding emphasizes the importance of the behaviour change process in promoting PF (Fletcher & Hayes, 2005), particularly for parents of children with ASD, enabling them to align their values and priorities and take actions that support both their children and their own well-being (Marino et al., 2021).

The concurrent validity results indicate that there are positive correlations between the Psy-Flex-C and the CompACT and its subscales. These findings suggest that the 6-item Psy-Flex-C could effectively assess PF similar to the CompACT, covering the six psychological aspects/processes of ACT. The Psy-Flex-C is significantly and positively correlated with the three subscales of the CompACT, namely, openness to experience, behavioural awareness, and valued action. These subscales align with the ACT triflex, which combines the six core ACT processes into three functional units: “open up (incorporated with defusion and acceptance)”, “be present (incorporated with self-as-context and contacting the present moment)”, and “do what matters (incorporated with values and commitment action)” (Harris & Hayes, 2019). Therefore, the Psy-Flex-C can be regarded as a reliable alternative instrument for assessing all aspects of ACT in a time-effective manner.

Satisfactory known-group validity of the Psy-Flex-C was obtained from the findings of significant differences in scores between the groups of parents with high and low parenting stress. This result was consistent with a cross-sectional study conducted by Lobato et al. (2022), the findings indicated a statistically significant correlation between higher levels of PF in parents/caregivers of children/adults with intellectual disabilities and lower levels of parenting stress. The possible reason for this correlation was that PF could act as a personal resource for effectively managing the negative experiences in performing the parenting role and responsibility. Parents with lower PF might negatively evaluate parenting stress and employ negative stress management strategies such as control, avoidance, or suppression (Whittingham & Coyne, 2019). However, these strategies could paradoxically amplify negative experiences, leading to higher stress levels and maladaptive parenting practices (Coyne et al., 2009). Another recent study in 250 mothers of children with ASD supported the link between lower PF and ineffective parental practices, as observed in a study by Fonseca et al. (2020), highlighting the importance of PF in promoting sensitive responses to children’s needs and positive parenting practices, even in the presence of parenting stress. However, future studies are recommended to focus on exploring the mechanisms through which PF influences parenting stress and strategies among these parents.

In addition, the convergent validity results indicate that the Psy-Flex-C is negatively associated with scores in parenting stress and its domains (PD, PCDI, and DC). These significant relationships further strengthen the evidence that these factors, such as negative psychological outcomes, parent‒child relationships, and children’s conditions, are related to PF in the parenting context. Additionally, the findings align with previous research suggesting that improving parents’ PF, particularly by focusing on reducing parenting stress and symptomatology (e.g., distress; Kulasinghe et al., 2021), could help parent‒child dyads improve their interaction and interrelationships and, thus, further support and improve their children’s neuro-developmental health conditions (Marino et al., 2021).

Several limitations are identified in this study, which deserve to be addressed in future studies. First, we did not include bilingual parents of children with ASD for the semantic equivalence test. Bilingual parents are recommended to be included in semantic equivalence testing to enhance translation consistency and prevent cultural biases between the original (English) and translated (Chinese) versions. Indeed, the practicability of this method for this study was limited by the availability of bilingual parents of children with ASD in mainland China. However, the authors mitigated this concern by inviting 21 Ph.D. students in nursing or psychology (mainly bilingual) to compare the original English and translated Chinese versions. Second, although participants were recruited from special education from three different provinces in mainland China, the convenience sampling method limited the generalizability of the findings. Therefore, further studies are recommended to use a random sampling method to recruit larger samples from wider geographic sites. Third, only 50 out of 248 parents were randomly selected to participate in the evaluation of test–retest reliability. Even though the results showed that the Psy-Flex-C had good test–retest reliability, future studies are encouraged to utilize a large sample size of people from diverse sociodemographic and clinical backgrounds, as well as people from different geographical regions. Moreover, further testing of the psychometric properties of the Psy-Flex is suggested to confirm its construct validity and its one-factor structure using diverse samples and settings of Chinese populations. For example, given that the ultimate goal of interventions targeting PF is to improve clients’/parents’ mental health and well-being (Stenhoff et al., 2020), future studies are suggested to examine the convergent validity of Psy-Flex with positive outcomes (e.g., quality of life, self-care) across diverse samples. Finally, due to the limitations of current measures designed specifically for assessing parental PF, this study selected a general context tool (Plex-Flex), and tested the psychometric properties of Psy-Flex-C among a specific parent population (parents of children with ASD). And to ensure the potential for generalizability in future studies, this study did not modify the content of Psy-Flex-C to the context of parenting or ASD. However, the sample in this study might not be representative to those divorce parents who are solely responsible for caring for their child with ASD, as well as those with multiple children with ASD. This might reduce the applicability of our findings to those specific subsets of parents who faced a heavier caregiving burden. Thus, future studies are recommended to tailor and validate this instrument within parenting and ASD context, encompassing diverse samples of parents to gain insights into their unique challenges, support needs, and family dynamics.

Despite the limitations noted above, the Psy-Flex-C can be useful for measuring the level of PF in Chinese parents of children with ASD. The questionnaire, which is simply phrased and user-friendly, took the participants approximately 5 min to complete. Therefore, the Psy-Flex-C could be more suitable than measurement instruments that require significant time, particularly for parents with heavy caregiving demands and responsibilities. Moreover, the Psy-Flex-C applies situational and temporal specifiers (i.e., past one week) to enhance its context sensitivity. This design helps to reduce the potential for recall bias and inaccuracies, which could potentially affect the validity results (e.g., test–retest validity) (Ong et al., 2019). With increasing demands for outcome evaluation (especially outcomes targeting parents’ mental health and well-being) of family support interventions (e.g., ACT or ACT-based interventions) for parents of children with ASD (Yu et al., 2019), measures of PF, such as the Psy-Flex-C in this study, should be important for developing and/or testing psychometrically sound/valid outcome measures of the level of PF in relevant research and clinical settings.

This study employed a rigorous process involving translation, expert comments, and online questionnaire surveys to evaluate and ensure that the Psy-Flex-C could be a reliable and valid measure among Chinese parents of children with ASD. Validating this scale is crucial for healthcare providers and researchers to comprehend the specific psychological construct (PF) of parents of children with ASD. To further investigate the validity, reliability and generalizability of the scale, researchers can test it with a wide range of populations across various practice settings, regions, and cultural backgrounds.

## Data Availability

All data generated or analyzed during this study are included in this published article as Supplementary information files.
